# The genomic diversification of grapevine clones

**DOI:** 10.1186/s12864-019-6211-2

**Published:** 2019-12-12

**Authors:** Amanda M. Vondras, Andrea Minio, Barbara Blanco-Ulate, Rosa Figueroa-Balderas, Michael A. Penn, Yongfeng Zhou, Danelle Seymour, Zirou Ye, Dingren Liang, Lucero K. Espinoza, Michael M. Anderson, M. Andrew Walker, Brandon Gaut, Dario Cantu

**Affiliations:** 1 Department of Viticulture and Enology, University of California Davis, Davis, CA 95616 USA; 2 Department of Plant Sciences, University of California, Davis, CA 95616 USA; 3 Department of Ecology and Evolutionary Biology, University of California, Irvine, CA 92617 USA

**Keywords:** Clonal propagation, DNA methylation, Genome diversification, Somatic mutations, Structural variation, Transposable elements

## Abstract

**Background:**

Vegetatively propagated clones accumulate somatic mutations. The purpose of this study was to better appreciate clone diversity and involved defining the nature of somatic mutations throughout the genome. Fifteen Zinfandel winegrape clone genomes were sequenced and compared to one another using a highly contiguous genome reference produced from one of the clones, Zinfandel 03.

**Results:**

Though most heterozygous variants were shared, somatic mutations accumulated in individual and subsets of clones. Overall, heterozygous mutations were most frequent in intergenic space and more frequent in introns than exons. A significantly larger percentage of CpG, CHG, and CHH sites in repetitive intergenic space experienced transition mutations than in genic and non-repetitive intergenic spaces, likely because of higher levels of methylation in the region and because methylated cytosines often spontaneously deaminate. Of the minority of mutations that occurred in exons, larger proportions of these were putatively deleterious when they occurred in relatively few clones.

**Conclusions:**

These data support three major conclusions. First, repetitive intergenic space is a major driver of clone genome diversification. Second, clones accumulate putatively deleterious mutations. Third, the data suggest selection against deleterious variants in coding regions or some mechanism by which mutations are less frequent in coding than noncoding regions of the genome.

## Background

Cultivated grapevines are clonally propagated. As a result, the genome of each cultivar is preserved, except for the accumulation of mutations over time that can generate distinguishable clones [1–4]. Somatic mutations are responsible for several notable phenotypes. For example, a single, semi-dominant nucleotide polymorphism can affect hormone response [5]. The presence or absence of the *Gret1* retrotransposon in the promoter of the *VvmybA1* transcription factor is associated with differences in the color of clones [6], as do additional mutations affecting the color locus [7–10]. The fleshless fruit of an Ugni Blanc clone and the reiterated reproductive meristems observed in a clone of Carignan are both caused by dominant transposon insertion mutations [11, 12]. In citrus, undesirable mutations can be unknowingly propagated that render fruit highly acidic and inedible [13, 14]. Interestingly, somatic mutations in plum are associated with a switch from climacteric to non-climacteric ripening behavior [15].

There is limited understanding and evidence of the extent, nature, and implications of the somatic mutations that accumulate in clonally propagated crops [16]. Genotyping approaches based on whole genome sequencing make it possible to identify genetic differences without predefined markers [17–19] and expedite learning the genetic basis of valuable traits and developmental processes [15, 20]. Still, few previous studies have used genomic approaches to study somatic variations among clones [17–21]. Carrier *et al.* (2012) found that transposable elements were the largest proportion of somatic mutation types affecting four Pinot Noir clones [18]. Whole genome sequencing was also used to study structural variations and complex chromosomal rearrangements in Tempranillo and to better understand the basis of somatic mutations giving rise to red versus white fruit, comparing diverse accessions of phenotypically distinct Tempranillo Tinto and Tempranillo Blanco [20]. Genomic tools could be used to comprehensively describe the extent of somatic mutations and infer the processes affecting clone genomes.

Mutations occur in somatic cells that proliferate by mitosis. These can occur by a variety of means, including single base-pair mutations [22, 23] that are more prevalent in repetitive regions because methylated cytosines passively deaminate to thymines [24–26], polymerase slippage that drives variable microsatellite insertions and deletions [27], and larger structural rearrangements and hemizygous deletions [10, 20]. Transposable elements are also a major source of somatic mutations in grapevines [18], though transcriptional and post-transcriptional mechanisms exist to prevent transposition and maintain genome stability [28–31]. Notably, methylation of transposable elements is one specific mechanism that prevents transposition.

At the cellular level, distinct clones can emerge following a mutation in a shoot apical meristem that spreads throughout a single cell layer, creating periclinal chimeras. This chimera is stable for Pinot Meunier, a clone of Pinot Noir with distinct L1 and L2 layers [3]. Each cell layer in a stratified apical meristem like that observed in grape [32] is developmentally distinct. Cell layers with distinct genotypes will remain so provided cell divisions occur anticlinally. But, periclinal divisions and cellular rearrangements can result in the homogenization of a mutant genotype across cell layers [33]. This is the case for green-yellow bud sports of the grey-fruited Pinot Gris, wherein sub-epidermal cells invaded and displaced epidermal cells that produce pigment in fruits [9]. In contrast to replacement (L1 cells invade L2), displacement is likely more common because of the relative disorganization of the inner cell layers [32, 33].

Meristem architecture is related to the fate of somatic mutations, as it influences the impact of these mutations and the likelihood of competition between cell lineages, also known as diplontic selection [34–36]. Provided each cellular layer is maintained by anticlinal divisions, deleterious mutations can be preserved in periclinal chimeras [35, 37]. The predominance of “hidden”, heterozygous recessive somatic mutations [2, 37] may also shield somatic mutations from selective forces. These factors are permissive of the accumulation of somatic mutations. Diplontic selection could occur if periclinal cell divisions result in the invasion of one cell layer by cells from another [34, 35]. This mechanism could oppose the accrual of deleterious mutations expected by Muller [38, 39]. Evidence of diplontic selection in plants is remarkably scarce [37], though its likelihood given different circumstances has been modeled [34, 35, 40]. Human action may also serve as a selective force, rejecting clones or individuals with mutations that manifest as undesirable traits. Selection may also occur at the level of the individual cell; cells with dominant deleterious mutations, haploinsufficiency-driven deleterious phenotypes, or any mutation made manifest by other means could be selected against and this might inhibit their spread throughout a single cell layer. Given the prevalence of chimerism and rearrangements documented in the model [9, 33], grapevine is suitable for investigating somatic mutation and the possibility of selection in vegetatively propagated plants.

Zinfandel is the third-most cultivated wine grape in California [41, 42]. DNA profiling produced evidence that Zinfandel is synonymous with Primitivo grown in Italy [43] and Croatian Pribidrag and Crljenak Kastelanski [44]. Historical records plus the cultivation of closely related cultivars support Croatia as the likely origin of Zinfandel [44–47] and also that Primitivo was likely brought to the Gioia del Colle region in Italy by Benedictine monks in the seventeenth century [3, 48]. The reported variability in Zinfandel [49–51], including subtle variability in phenolic metabolites (Additional file 1), and its long history of cultivation make it a useful model for studying clonal variation in grapevine, specifically, and the nature of the accumulation of somatic mutations in clonally propagated crops, generally.

The purpose of this study was to better understand the nature of the somatic variations that exist among grapevine clones grown exclusively under a regime of vegetative propagation. Representatives of at least a portion of Zinfandel’s history [44–47] from Croatia, Italy, and California were sequenced and compared using Zin03 as reference (Table 1). First, we show that intergenic space drives clonal diversification. As previously reported for Pinot Noir, transposable element insertions varied among clones [18]. This report expands that understanding to implicate methylation as an indirect driver of clonal diversification. Somatic heterozygous Single Nucleotide Variants (SNVs) that occurred in few or individual clones were most observed in repetitive intergenic regions. This is likely because of the high levels of transposition-inhibiting methylation and associated transition mutations that are prevalent there. Second, the data support an important component of Muller’s ratchet [38], that asexually propagated organisms accumulate deleterious mutations. Third, somatic mutations were relatively scarce in the coding regions of genes relative to introns and intergenic space, suggesting some mechanism by which deleterious mutations are less common there.

**Table 1:** Clone identifying information

Clone #	Common name	Origin	Foundation Plant Services
1	Primitivo	Bari, Italy	Primitivo FPS 03
2	Primitivo	Conegliano, Italy	Primitivo FPS 06
4	Pribidrag	Svinšće, Croatia	Zinfandel FPS 43.1
5	Pribidrag	Svinšće, Croatia	Zinfandel FPS 44.1
6	Zinfandel	California, USA	Zinfandel FPS 10
7	Zinfandel	California, USA	Zinfandel FPS 24
8	Zinfandel	California, USA	Zinfandel FPS 37
9	Zinfandel	California, USA	Zinfandel FPS 39
10	Zinfandel	California, USA	Zinfandel FPS 56.1
11	Zinfandel	California, USA	Zinfandel FPS 40
12	Pribidrag	Marušići, Croatia	In testing at FPS
13	Pribidrag	Svinšće, Croatia	Mother of FPS 43.1
14	Crljenak kaštelanski	University of Zagreb, Croatia	–
15	Pribidrag	Svinšće, Croatia	Mother of FPS 44.1
Zin03	Zinfandel	California, USA	Zinfandel FPS 03

## Results

### Zinfandel genome assembly, annotation, and differences between haplotypes

The clone used for the genome assembly, Zinfandel 03 (Zin03), was acquired by FPS in 1964 from the Reutz Vineyard near Livermore, California that was planted during Prohibition (1920–1933) [52]. Zin03 was sequenced using Single Molecule Real-Time (SMRT; Pacific Biosciences) technology at ~98x coverage and assembled using FALCON-unzip [53], a diploid-aware assembly pipeline. The genome was assembled into 1509 primary contigs (N50 = 1.1 Mbp) for a total assembly size of 591 Mbp, similar to the genome size of Cabernet Sauvignon (590 Mbp) [53] and larger than Chardonnay (490 Mb) [19] and PN40024 (487 Mb) [54]. Fifty two percent of the genome was phased into 2246 additional sequences (haplotigs) where the homologous chromosomes were distinguishable with an N50 of ~ 442 kbp (Table 2). A total of 53,560 complete protein-coding genes were annotated on the primary (33,523 genes) and haplotig (20,037 genes) assemblies (Table 2).

**Table 2:** Summary statistics of the Zinfandel genome assembly and annotation

	Primary	Haplotig
Total length	591,171,721	306,029,957
Number of contigs	1509	2246
N50	1,062,797	442,393
N75	366,308	185,785
L50	154	200
L75	395	463
Median contig length (bp)	161,249	37,307
Longest contig (bp)	7,901,503	2,609,171
Shortest contig (bp)	17,787	1970
Average GC content (%)	34.45%	34.37%
Number of genes	33,523	20,037
	*Total*	*Average per gene*
Number of exons	244,880	4.57
Number of introns	191,320	3.57
	*Average (bp)*	*Maximum (bp)*
mRNA lengths	4166	94,143
Exon lengths	245.79	7992
Intron lengths	191,320	41,647
Intergenic distances	10,309	302,473

Of the 20,037 genes annotated on the haplotig assembly, 18,878 aligned to the primary assembly, leaving 1159 genes that may exist hemizygously in the genome due to structural variation between homologous chromosomes or because of substantial divergence in sequence between haplotypes. These genes were annotated with a broad variety of putative functions and included biosynthetic processes, secondary metabolism, and stress responses. Long reads were mapped to both the primary and haplotig assemblies to evaluate the circumstances that explain the differences between haplotypes. Structural variants (SVs) between the haplotypes were examined by mapping long SMRT sequencing reads onto Zin03 with NGMLR and calling SVs with Sniffles [55]. As the most contiguous assembly, reads were mapped to the Zin03 primary assembly to examine genome-wide structural variations that may occur between haplotypes. In addition, reads were mapped to the haplotigs specifically to see whether structural variations could account for the genes uniquely present in the haplotigs.

**Fig. 1:**
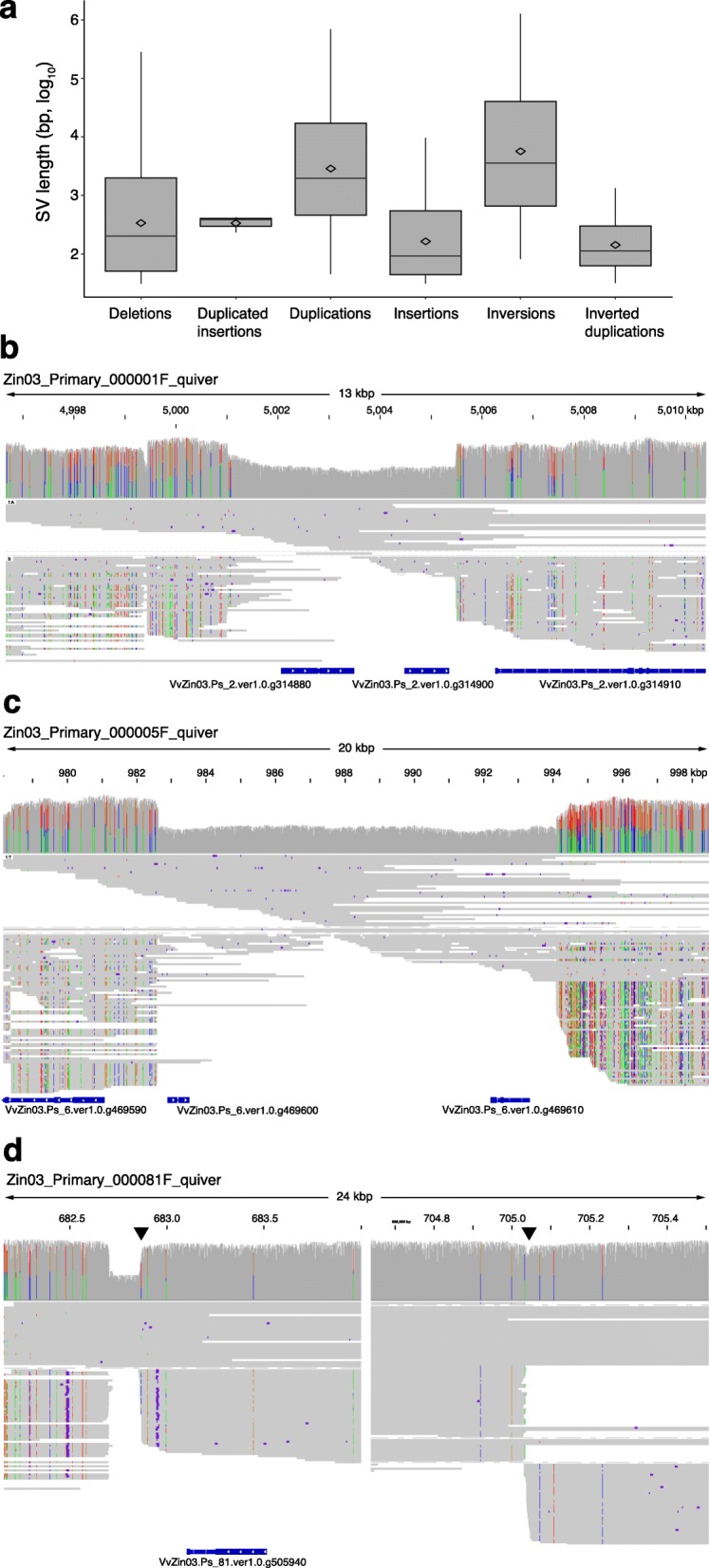
Structural variation between Zin03 haplotypes. **a**. Distribution of structural variation sizes. Boxplots show the 25th quartile, median, and 75th quartile for each type of SV. Whiskers are 1.5^Inter-Quartile Range^. Diamonds indicate the mean log_10_(length) of each type of SV; **b,c,d.** Examples of heterozygous structural variants between haplotypes that intersect genes. For each reported structural variation, (from top to bottom) the coverage, haplotype-resolved alignment of reads, and the genes annotated in the region are shown; **b.** 4 kbp heterozygous deletion of two genes; **c.** 11 kbp heterozygous deletion of two genes; **d.** 22 kbp inversion that intersects a single gene. Triangles indicate boundaries of the inversion. A gap is shown rather than the center of the inverted region

A total of 22,399 SVs accounted for 6.94% (41.0 / 591 Mbp) of the primary assembly’s length and 6.02% (8.4 / 139 Mbp) of the primary assembly’s gene-associated length (Fig. 1, Table 3). SVs intersected 4559 genes in the primary assembly (13.6% of primary assembly genes) and 390 SVs spanned more than one gene. The long reads aligned to the primary assembly support that large, heterozygous deletions and inversions occurred in the Zin03 genome that were either inherited from different structurally distinct parents or arose during clonal propagation (Fig. 1 b,c,d). Importantly, there was substantial hemizygosity in the genome, with long reads supporting deletions affecting 2521 genes and 4.56% of the primary assembly’s length (Table 3).

**Table 3:** Sniffles analysis of structural variation between Zinfandel parental haplotypes and between Zinfandel and Cabernet Sauvignon

	Zinfandel SV relative to Zinfandel primary assembly					Cabernet Sauvignon SV relative to Zinfandel primary (P) assembly and haplotigs (H)				
	*Median Size (bp)*	*Count*	*Genes*	*Total SV size (Mb)*	*% Primary assembly*	*Median Size (bp)*	*Count*	*Genes*	*Total SV size (Mb)*	*% genome (P + H)*
Deletions	203	12,031	2521	26,953,558	4.56	196	P: 34,259	6761	87,430,736	9.74
							H: 12,104	2458	27,582,275	3.07
Duplications	1966	553	535	7,604,041	1.29	5518	P: 2264	2787	41,289,418	4.60
							H: 620	499	7,445,635	0.83
Insertions	92	9647	2081	5,594,259	0.95	88	P: 28,825	3708	19,869,958	2.21
							H: 8582	1517	4,000,833	0.45
Inversions	3592	111	391	5,521,214	0.93	6037	P: 517	1305	18,814,293	2.10
							H: 90	135	1,862,657	0.21
Duplicated Insertions	385	3	2	6861	0.0012	339	P: 6	0	42,698	0.0048
							H: 3	2	1223	0.0001
Inverted Duplications	113	54	11	12,930	0.0022	293	P: 51	9	32,283	0.0036
							H: 14	3	9534	0.0011

Next, we considered whether specific structural variation could account for the 1159 genes uniquely found in the haplotig assembly. Three hundred eighty-two genes of the previously mentioned 1159 genes that uniquely exist within the haplotig assembly intersected structural variations. Two hundred ninety of these intersected deletions, accounting for the failure to identify them on the primary assembly. Some of the haplotig genes that failed to map to the primary assembly intersected additional types of SVs, including duplications (80 genes), insertions (89 genes), and inversions (16 genes).

These results reveal structural differences between Zinfandel’s haplotypes. These differences could have been inherited and/or could be somatic mutations. Overall, these structural variations affected 4559 primary assembly genes (Additional file 2). These genes were associated with 27 cellular components, 28 functional GO categories, and 50 biological processes (Additional file 2). Some of the most common biological processes associated with these genes were catabolic process (351), response to stress (259), biotic stimulus (263), carbohydrate metabolism (259), and secondary metabolism (120). The most abundant functional categories represented included hydrolase activity (648), kinase activity (146), protein binding (144), transport (134), transcription factor activity (156), and signaling receptor activity (33).

### Differences in structure and gene content between Zinfandel and Cabernet Sauvignon

**Fig. 2:**
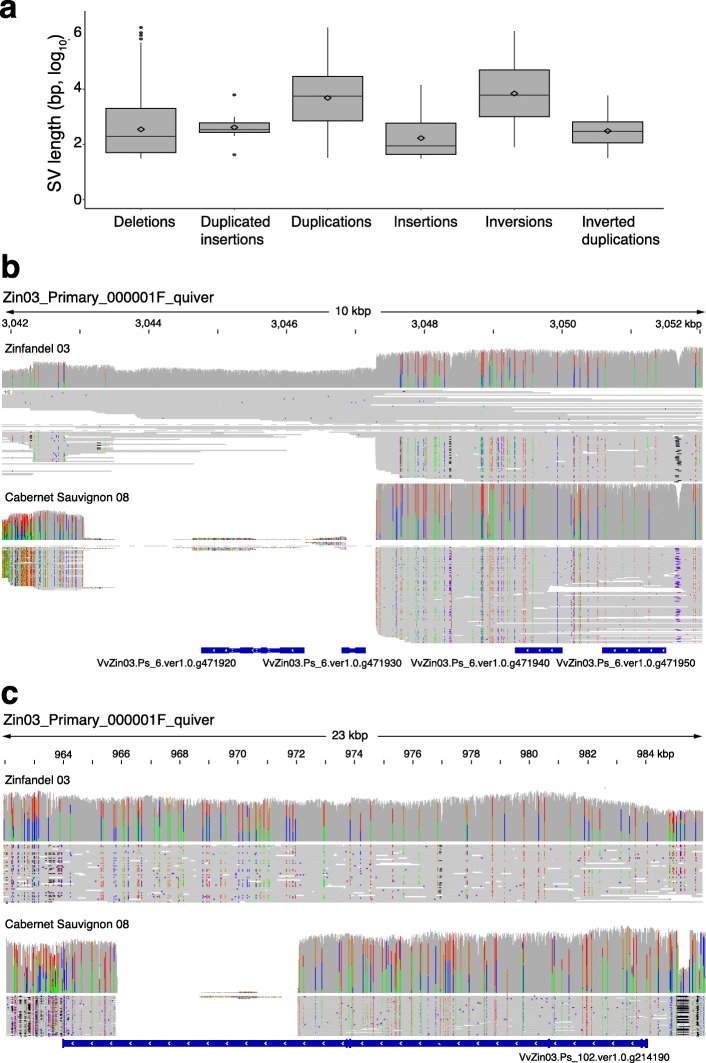
Gene content and structural variability between Zin03 and Cabernet Sauvignon. **a.** Distribution of structural variation sizes. Boxplots show the 25th quartile, median, and 75th quartile for each type of SV. Whiskers are 1.5^Inter-Quartile Range^. Diamonds indicate the mean log_10_(length) of each type of SV; **b,c.** Selected deletions in Cabernet Sauvignon relative to Zin03 that intersect genes. For each reported deletion, (from top to bottom) the coverage of reads over the region by long Zinfandel and Cabernet Sauvignon reads, haplotype-resolved alignment of the reads, and the genes annotated in the region are shown; **b.** Two genes are completely deleted in Cabernet Sauvignon relative to Zinfandel and are deleted in one Zinfandel haplotype; **c.** One gene contains a homozygous partial deletion in Cabernet Sauvignon

The Zin03 genome was compared to Cabernet Sauvignon (CS08) to assess how Zin03 gene content differs from Cabernet Sauvignon. CS08 was recently used to construct the first diploid, haplotype-resolved grape genome for which long reads are available [53]. We identified 576 genes present in Zin03 that were not present in CS08. Structural differences between Zin03 and CS08 were explored in more detail by mapping the long SMRT reads of CS08 onto Zin03’s primary and haplotig assemblies with NGMLR and calling SVs with Sniffles (Fig. 2a, Table 3). Overall, these SVs corresponded to 17.74% (159/ 897 Mbp) of the Zin03 assembly’s total length, 12.5% of its total protein-coding regions (28 / 223 Mbp), and 25.6% of all Zin03 genes. SVs affected 9885 genes in the primary assembly and 3804 genes in the haplotigs. Some genes intersected more than one structural variation. The long CS08 reads aligned to Zin03’s primary assembly support that large SVs exist between the two genotypes (Fig. 2b, c). Next, we considered whether specific structural variation called by Sniffles could account for 576 Zin03 genes absent from CS08 identified by mapping Zin03 genes to CS08. Of these 576 Zinfandel genes, 268 genes intersected 454 deletions supported by long CS08 reads aligned to Zin03.

High levels of structural variation between Zinfandel (Zin03) and Cabernet Sauvignon (CS08) were observed and these affected considerable protein-coding regions of the genome. These results justify constructing a Zinfandel-specific reference to better capture genomic variability among Zinfandel clones that could otherwise be missed, particularly if an alternative reference lacks sequences present in Zinfandel.

### Relatedness among Zinfandel clones

Fifteen Zinfandel clones, including Zin03, were sequenced using Illumina. The resulting reads were aligned to the Zin03 primary assembly to characterize SNVs, small insertions and deletions (INDELs), variable transposon insertions, and large structural variants. The validity of these calls were evaluated genome-wide and for several selected variants. Greater than 90% of the heterozygous SNVs called by GATK for Zin03 relative to the Zin03 primary assembly were also called by Mummer and/or Clairvoyant when comparing the primary assembly and haplotigs (Additional file 3: Table S1). Ten selected variants were also confirmed (~ 80%) by Sanger sequencing (Additional file 3: Table S2). Though a substantial number of variants were reproducible by one or two other methods, the absolute number of variants reported in this study is possibly inflated.

**Fig. 3:**
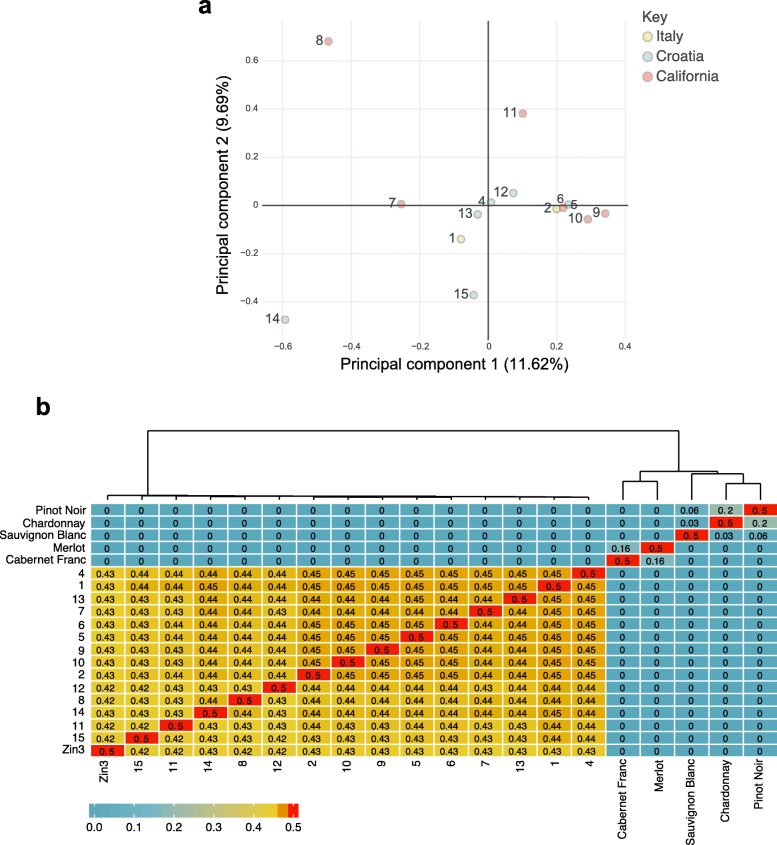
The relationships between Zinfandel selections based on SNVs and sites at which all clones were called by GATK **a.** Principal component analysis of Zinfandel selections based on SNVs. Zin03 was not included in the analysis; **b.** Kinship analysis of Zinfandel selections and other cultivars with known relationships. The Kinship coefficient, PHI, is shown, as well as a dendrogram constructed by hierarchically clustering genotypes using their kinship coefficients

Principal Component Analysis (PCA) of variants among the clones showed no clear pattern in their relationships to one another based on their recorded origins prior to acquisition (Fig. 3a). The ambiguity of the clones’ histories means that it should not be taken for granted that the Californian selections, for example, ought to be more closely related to one another than to the Italian or Croatian selections. Unique clonal SNVs could further obscure their relationships.

Interestingly, Pribidrags 5 and 15 do not co-localize in the PCA (Fig. 3a, Table 1). There are only two pairs of clones whose relationship to one another is known. Pribidrag 15 was a cutting from the mother of Pribidrag 5; Pribidrag 13 was a cutting from the mother of Pribidrag 4. Pribidrags 4 and 5 were both subjected to microshoot tip tissue culture therapy (Table 1). However, the complete lineages of these pairs and the other clones prior to their introduction to curated collections is unknown. The process of tissue culture may have introduced mutations to the clones in an inconsistent manner, such that Pribidrag 4 appeared more closely related to its mother than Pribidrag 5. Note, the percent alignment of Pribidrag 15 reads to Zin03 (80%) was also markedly lower than the other clones (> 94%); this technical difference may have contributed to the distance between this pair as well (Additional file 4: Table S1).

A kinship analysis [56] was then used to quantitatively assess the relationships between the Zinfandel selections. These values range from zero (unrelated) to 0.5 (self). Additional cultivars were included in the analysis with known relationships to help contextualize the differences between clones and evaluate the integrity of the analysis (Fig. 3b). Cabernet Franc and Merlot have a parent - offspring relationship, as do Pinot Noir and Chardonnay [57, 58]. These pairs had kinship coefficients of 0.16 and 0.20, respectively (Fig. 3b). As a possible grandparent of Sauvignon Blanc, Pinot Noir had a kinship coefficient of 0.06 with Sauvignon blanc [59, 60]. Zinfandel selections had kinship coefficients between 0.42 and 0.45; this is likely because of the accrual of heterozygous somatic mutations among clones (Fig. 3b).

Somatic mutations in clones are expected to be heterozygous. Across the Zinfandel clones, the median number of homozygous and heterozygous variants called relative to Zin03 were 42,869 and 710,080, respectively (Additional file 4: Table S2). On average, 5.68% of variant sites called did not share the Zin03 reference allele. Like non-reference calls for Zin03 mapped to itself, homozygous non-reference calls among clones are likely errors. It also does not appear that tissue culture influenced the number of heterozygous variants present (Mann-Whitney test, *p-value* > 0.1, Additional file 4: Table S2).

### Clonal versus cultivar genetic variability

On average, 6,153,832 variant sites (heterozygous plus homozygous) were identified in other cultivars (Pinot noir, Chardonnay, Sauvignon Blanc, Merlot, Cabernet Franc) relative to Zin03 (Additional file 4: Table S2). Both of these figures exclude heterozygous sites at which the diploid genotype called for a given sample was identical to that called for Zin03.

**Fig. 4:**
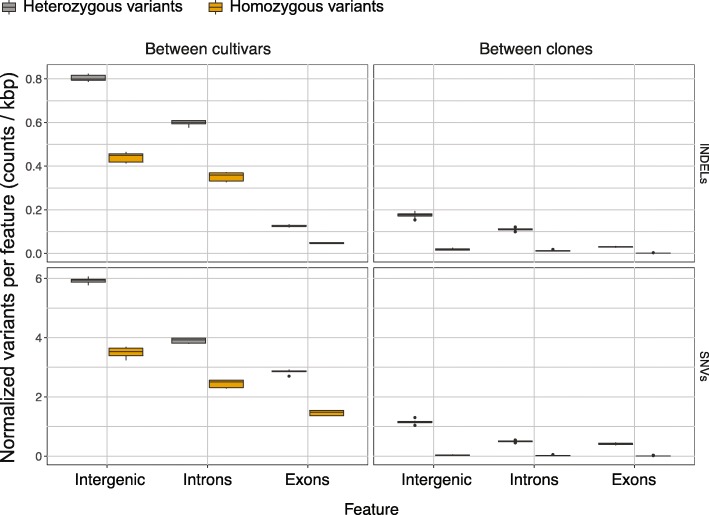
Characterization of variants and their frequency among Zinfandel selections and other *vinifera* cultivars (Pinot Noir, Chardonnay, Merlot, Cabernet Franc, and Sauvignon Blanc). Only variant sites at which all samples were called by GATK (All non-Zinfandel clones and Zin03, left-hand column; All clones, right-hand column) were used. The normalized rate of variants (number of variants divided by the total feature length in the genome * 1 k) by type (SNV, INDEL), feature (Intergenic, Intron, Exon), and genotype (Non-Zinfandel cultivars, Zinfandel clones). Boxplots show the 25th quartile, median, and 75th quartile

Considering only sites at which all non-Zinfandel cultivars were called and where all Zinfandels were called, variants were 8.2X more frequent in other cultivars relative to Zin03 than for Zinfandel clones; on average, variants in clones occurred once every 971 bases and once every 116 bases in other cultivars (Additional file 4: Table S3). However, the ratio of transitions to transversion mutations and the proportions of the predicted variant effects were similar for both groups (Additional file 4: Table S3). The normalized count of variants differed between cultivars and Zinfandel clones on the basis of variants’ location in the genome, the type of variant, and the zygosity of the variant (Fig. 4).

Variants in non-Zinfandel cultivars and heterozygous variants among Zinfandel clones were significantly more prevalent in intergenic space than in introns and exons and significantly more prevalent in introns than exons (Tukey HSD, *p <* 0.01). As expected, homozygous variants between cultivars were substantially more abundant than homozygous variants among clones (Fig. 4, Additional file 4: Table S2). The low levels of homozygous variants observed among clones are likely technical errors that may have arisen during variant calling over hemizygous regions and/or large regions with high sequence divergence between haplotypes.

**Fig. 5:**
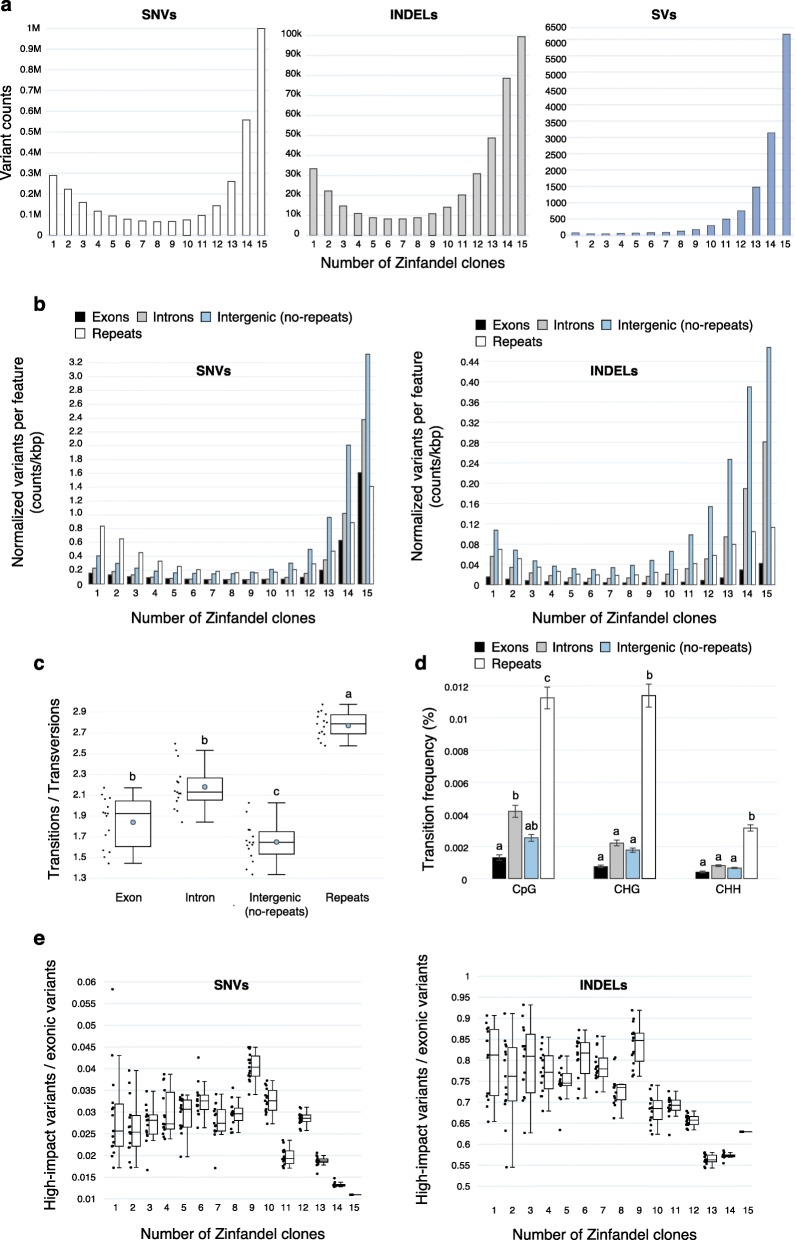
The abundance and impact of shared and unique heterozygous mutations among Zinfandel clones. Only loci at which all clones were called by GATK were used. **a.** The number of heterozygous SNVs, INDELs, and SVs shared by only N Zinfandel clone(s); **b.** The number of SNVs and INDELs shared by only N clone(s) in exons, introns, intergenic repeats (“Repeats”), and non-repetitive intergenic space; **c.** The ratio of transitions (Tr) to transversions (Tv) for heterozygous SNVs that uniquely occur in single Zinfandel clones and in different genome features. Different letters indicate significant differences in Tr/Tv rates between features (Tukey HSD, *p* < 0.01). The mean is shown as a blue circle; **d.** The mean percentages of CpG, CHG, and CHH in exons, introns, intergenic repeats (“Repeats”), and non-repetitive intergenic space that experience transition mutations. Standard error is shown. Heterozygous SNVs that uniquely occur in a single Zinfandel clone were used. Different letters indicate significant differences (Tukey HSD, *p* < 0.01); **e.** Proportion of exonic SNVs and INDELs that are putatively deleterious and shared by only N Zinfandel clone(s)

The accrual of predominantly heterozygous and likely recessive variants [2] is consistent with what would be expected given physically separate homologous chromosomes and the absence of sexual reproduction. The differences in mutation frequency in different features were initially surprising; if somatic mutations occurred randomly and absent mechanisms that make certain sites more or less susceptible to mutation, then different regions of the genome should have had equal normalized rates of mutations. This was not the case (Fig. 4, Fig. 5).

### The accrual of somatic mutations in Zinfandel clones

Heterozygous sites found among the 15 Zinfandel clones ought to be a mixture of sites inherited from their shared ancestral plant and somatic mutations that arose during clonal propagation. Thirty percent of heterozygous SNVs, 24% of heterozygous INDELs, and 47% of heterozygous structural variants were shared by all 15 Zinfandel clones (Fig. 5a). These are the heterozygous sites inherited from Zinfandel’s parents and this result is consistent with the derivation of these clones from a common ancestral mother plant.

Individual and subsets of Zinfandel clones accumulated heterozygous mutations (Fig. 5a). Thirteen percent and 16% of heterozygous INDELs and SNVs, respectively, and 1% of large (> 50 bp) structural variants occurred in only one or two clones (Fig. 5a). The interesting shape of the distribution shown in Fig. 5a was reproducible when heterozygous SNVs called by GATK and Clairvoyant, GATK and Mummer, or at least two of these tools were used to replot the figure (Additional file 3: Figure S1). The median number of unique heterozygous SNVs was not significantly different between tissue-cultured samples and clones not subjected to tissue-culture (Mann-Whitney test, *p* > 0.5, Additional file 4: Table S2).

The distribution of SVs called by Delly was slightly different than those of SNVs and INDELs (Fig. 5a). For both SNVs and INDELs, there were 3 and 3.5-fold as many heterozygous variants shared by all 15 clones as there were uniquely occurring variants; there were 71.5-fold more structural variants shared by all clones than there were unique variants in individual clones (Fig. 5a). This might imply that the mechanisms that give rise to or permit small mutations are more common among clones than large-scale SVs.

The distribution of unique and shared heterozygous INDELs in exons, introns, repetitive, and non-repetitive intergenic spaces were not equal (Fig. 5b). The distribution of INDELs in exons was significantly different than the distributions of INDELs in each other feature considered (Kolmogorov-Smirnov Test*, p* < 0.01). Similarly, the distribution of SNVs in genic (exons, introns) and intergenic (repetitive, non-repetitive) regions were not equal (Fig. 5b).

SNVs shared by all clones were most common in intergenic non-repetitive regions and introns and least common in exons and repetitive intergenic regions (Fig. 5b). Unique heterozygous SNVs occurred at high rates in repetitive intergenic regions (Fig. 5b). That shared heterozygous sites are mostly in non-repetitive intergenic space and unique heterozygous sites are mostly in repetitive space may have to do with the increased likelihood that methylated cytosines spontaneously deaminate and the prevalence of methylated repetitive sequences in those regions [22, 25, 29, 30]. This is also supported by the significantly higher ratio of transitions to transversions in repetitive intergenic regions than in exons, introns, and non-repetitive intergenic space (Fig. 5c). Furthermore, the mean percentage of CpG, CHG, and CHH sites affected by transition mutations was significantly higher in repetitive intergenic space than genic and non-repetitive intergenic spaces (Fig. 5d; Tukey HSD, *p* < 0.01). The mean percentage of CpG sites affected by transition mutations was also significantly higher in introns than exons (Tukey HSD, *p* < 0.01). Compatible with this hypothesis, INDELs, which should not increase in frequency due to methylation, did not occur preferentially in repeats (Fig. 5b). Interestingly, heterozygous SNVs shared by most clones (8 < *x* < 15, Fig. 5b) were less common in repetitive regions than in other features.

The impact of specific variants also varied with their prevalence among the clones (Fig. 5e). “High impact” mutations were predicted by SNPEff [61]. The high impact mutations identified in these data included exon losses, start and stop site gains and losses, frameshifts, gene fusions, splice acceptor mutations, and splice donor mutations. These mutations are predicted to be deleterious because of their disruptive effects on the coded protein. For these reasons, we designated such mutations as putatively deleterious in this manuscript. These were counted for each Zinfandel clone relative to Zin03. Relatively low proportions of heterozygous variants shared by all Zinfandel clones were putatively deleterious. In contrast, larger proportions of exonic SNVs and INDELs that occurred in individual or subsets of clones were putatively deleterious (Fig. 5e).

Together, these results show that somatic mutations are most numerous outside of coding regions of the genome. Clone genomes diversify most rapidly in the intergenic space, particularly in repetitive and likely methylated regions (Fig. 5). Though a minority of somatic mutations occurred in exons (Fig. 5b), we show that larger proportions of exonic mutations that occur in few or individual clones are deleterious than exonic heterozygous variants shared by all or most clones (Fig. 5e). In other words, clones accumulate putatively deleterious heterozygous mutations.

### Zinfandel clones incur unique transposon insertions

**Fig. 6:**
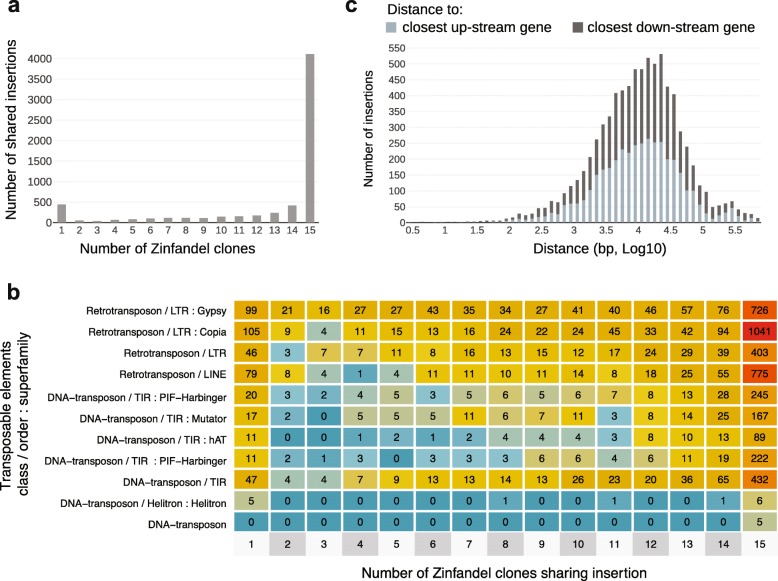
Transposable element insertions among Zinfandel selections. **a.** Transposable element insertions shared by only N Zinfandel selection(s) relative to PN40024; **b.** Types of transposable element insertions shared by only N Zinfandel selection(s) relative to PN40024; **c.** The proximity of intergenic transposable element insertions to PN40024 genes

Transposable element insertions (TEI) contribute to somatic variation in grape [6, 11, 12, 18]. Relative to PN40024, 6340 TEI were identified among the Zinfandel clones. A small fraction of TEI (6.94%) occurred uniquely in individual clones (Fig. 6a) and included 329 retrotransposons, mostly Copia and Gypsy LTRs, and 111 DNA-transposons (Fig. 6b). The majority (64.8%) of TEI were shared among the 15 Zinfandel clones. Five hundred thirty TEI occurred in only one, two or three clones (Fig. 6a). This result supports the derivation of these selections from a common ancestral plant and the accumulation of somatic variations over time. This pattern is also consistent with other types of somatic mutations (Fig. 5a).

In addition to being suggestive of their shared heritage, the positions of these insertions and their proximity to coding genes were notable. 2355 TEI were observed in 1622 coding genes. The remaining 3985 TEIs were in intergenic regions (Fig. 6c). The median upstream and downstream distance of intergenic TEs from the closest feature were 10,649 and 11,602 base-pairs, respectively. Twenty-five percent of TEI were within approximately 4 k bases of the closest feature (Fig. 6c).

## Discussion

Consideration of the genomic differences among Zinfandel clones revealed what is likely a complex history. Analyses of the relationships between clones did not reveal groupings of clones per their recorded countries of origin. There is very limited information about specific clonal lineages prior to their acquisition by FPS (http://fps.ucdavis.edu/fgrdetails.cfm?varietyid=1634). Somatic mutations may help identify individual clones but could also blur the historical relationships between them. It is also plausible that pairs of clones from any given region are not direct cuttings of one another but of Zinfandels from another region; the clones now grown in California, for example, may have been imported on independent occasions from various other regions, meaning some may indeed be more closely related to one of the Primitivo or Croatian clones than they are to other Californian clones.

There are costs and benefits associated with clonal propagation [16]. Among the benefits is that clonal propagation generally fixes heterozygous loci and valuable phenotypes. Despite the ambiguity of their lineage, the examination of SNVs, INDELs, transposable elements and other structural variants all support the derivation of the clonal selections from a common ancestral Zinfandel mother plant and show the accumulation of somatic mutations in individual and subsets of clones (Figs. 5 and 6). The structure of the Zinfandel genome, location of mutations among clones, their frequency and prevalence, and the relationship between these factors provides some insight into the nature of mutations in clonally propagated plants. Mutations among clones were predominantly heterozygous (Fig. 4) and larger proportions of heterozygous mutations in exons were putatively deleterious when shared by individual or a subset of clones (Fig. 5e). The increase in the proportion of deleterious alleles supports Muller’s ratchet, which posits that sex is advantageous and that clonal propagation increases mutational load [38].

Some unexpected observations were recorded. The abundance of SNVs in different features varied with the number of clones among which they were shared. In Fig. 5b, the values at *x* = 15 provide different information than those at *x* < 15. The values at *x* = 15 reflect the genetic distance between Zin03’s parents and the amount of sequence diversity between them for each feature, whereas the values at *x* < 15 show the changes over time in a clonally propagated cultivar. We might expect differences in sequence diversity for different types of features in the genome, generally [62], that are not necessarily identical to the pattern of somatic mutation accumulation. Though transition mutations in repeats are the most common unique SNVs, why highly shared heterozygous SNVs (8 < *x <* 15) are less abundant in repeats than in other features is not clear (Fig. 5b). As these SNVs are shared by many clones, they would be relatively older (versus those SNVs shared by < 8 clones), but it is not clear why the age of an SNV would be related to where it occurs in the genome.

Furthermore, we observed slight differences in the pattern of somatic mutation accumulation between SNVs, INDELs, and SVs, specifically an uptick in the number of unique somatic heterozygous sites at *x* = 1 for SNVs and INDELs that was less apparent for SVs (Fig. 5a). Although there may indeed be a biological basis for this, it is also plausible that technical differences in variant calling methods between GATK and Delly contribute to this subtle difference. Nonetheless, both sets of analyses support the diversification of clonally propagated grapevines derived from a common mother.

The set of variants identified by this work may serve as a primer for studies relating Zinfandel clones’ genotypes and phenotypes. Although the procedure used to test the validity of variants called was relatively successful and the distribution of unique and shared SNVs was reproducible (Additional file 3: Figure S1), several limitations of this study’s methods restrict what can be gathered about somatic variation in Zinfandel. The results of this procedure suggest that the absolute numbers of SNVs identified, including in repetitive regions, are possibly inflated. Even so, the ratio of transitions to transversions per feature type (Fig. 5c) is not likely a result driven by the magnitude of variants called within each feature. We do not have reason to think that the validity of variant calls would differ between exon, intron, and non-repetitive intergenic space. If variant calls were inflated in repeats to a degree greater than that in other features, this could affect the rates reported in Figs. 4, 5b, and d. Thus, the pattern observed should be regarded with greater confidence than the specific rates observed.

The greater abundance of SNVs observed here than by others [17, 19] may be partly biological and partly technical, with the latter being driven by the objectives of the study. The application of stringent filters enabled Roach et al. [19] to identify a small set of less than 2 k markers with which to reliably distinguish different Chardonnay clones, substantially fewer than the ~ 350 k SNVs shared by <= 14 Zinfandel clones and validated here with at least two independent bioinformatic tools and datasets (Additional file 3: Figure S1). These differences are likely technical in nature. In contrast, our results are far more comparable to those reported in a study of three Nebbiolo clones that used a similar analytical approach [17]. Gambino et al. reported between ~ 4.4 k and 8.5 k unique SNVs per clone. We report between 13.5 k and 30.7 k unique SNVs per clone, with a median of ~ 17.6 k (Additional file 4: Table S2). This modest disparity may be influenced by the cultivar and clones used; besides any effect of sample size on the number of unique variants identified per clone, the genetic distances between their Nebbiolo clones and between our Zinfandel clones are not necessarily the same.

Additional work should be undertaken to confirm specific SNVs, INDELs, TEs, and SVs among clones and to establish whether they contribute to differences among Zinfandel clones for an array of traits more expansive than those considered here. Furthermore, only up to two alleles were considered here despite the commonality of chimerism in grape. This study did not consider differences between cell layers or the pervasiveness of given variants within cell layers, but our understanding of somatic mutations and their fates would benefit from such accounting. Also, we observed considerable structural variation and hemizygosity in the Zinfandel genome. By calling somatic variants relative to the primary assembly using short reads, clonal variations in large regions of sequences represented only in the haplotigs were not considered in this study given this strategy and the tools used.

These and previous data do not tell which mutations are actually recessive or dominant, but they are expected to be largely heterozygous and recessive [2, 63]. This is why the variable normalized mutation abundance in exons, introns, intergenic space, and repeats is particularly interesting. The rarity of mutations in exons and commonality of mutations in repetitive intergenic space may have at least two components.

Mutations are likely more frequent in repetitive intergenic space as a result of the regulation of transposition by DNA methylation. Repetitive intergenic space had the highest rate of relatively unique SNVs, the ratio of transitions to transversions was significantly higher there than in other regions, and the portion of CpG, CHG, and CHH that incur transition mutations was highest in repeats. DNA methylation is an important epigenetic control and is one mechanism that maintains genome stability and impairs the transposition of mobile elements [29, 64, 65]. Methylated cytosines, however, spontaneously deaminate faster than unmethylated cytosines [24, 30]. Together, the prevalence of transposable elements and methylation present in the region account for the high rate of clonal SNVs in repetitive intergenic space. Also notable, these data show that some transposable elements are not entirely silenced, with a substantial number inserting in genes or in close proximity to genes (Fig. 6c). These insertions could be effectively inconsequential or not; transposable element insertions can result in novel transcripts and affect gene expression regulation [11, 66]. Gene body methylation is appreciated as a mutagenic “double-edged sword” [67], with benefits coming at the price. Recent work observed region-specific methylation in vegetatively propagated Sardinian white poplar that may serve an advantageous function [68] and others have suggested that the epigenome contributes to the success of vegetatively propagated plants [69]. Future work might also consider the long-term price associated with intergenic mutagenesis.

The rarity of exonic mutations was surprising. After accounting for the length of these spaces in the genome, we expected uniform rates of mutation accumulation in exons, introns, and non-repetitive intergenic space. Instead, we observed that mutations in exons were scarce and that relatively large fractions of heterozygous variants in individual and small subsets of clones were deleterious. Some degree of negative selection against deleterious variants in coding regions could explain why mutations were less frequent in coding than noncoding regions of the genome, but the mechanism by which this might occur remains an open question. The structures of apical meristems [35, 70] and the tendency of somatic mutations to be heterozygous and recessive [2] (and as a result, “hidden”) place constraints on the likelihood that deleterious mutations would be subjected to negative selection. The possibility of diplontic, clonal selection or competition between cell lineages that could purge otherwise consequential deleterious mutations has been modeled, but evidence of its occurrence is sparse [16, 34, 39]. Periclinal divisions across cell layers could enhance diplontic selection [34] against dominant and/or hemizygous recessive alleles. Even in the absence of exchange between cell layers, selection against cells carrying deleterious mutations could occur. Furthermore, we cannot discount the impact of human action; any mutations that gave rise to undesirable phenotypes would be selected against and excluded from subsequent propagation. Four and one half percent of Zinfandel’s genome is hemizygous; structural variations identified within the Zinfandel genome and the rampant hemizygosity reported in Chardonnay [10] could also expose otherwise hidden somatic variations to selective pressure hostile to the accumulation of deleterious mutations. Mutations that cause haploinsufficiency might also be exposed to purifying selection. In addition to selection, lower levels of methylation could also make exonic mutations less likely. Additional work should explore to what degree each of these factors, or others not considered here, explain why somatic mutations in exons were relatively infrequent and characterize the realized long-term consequences of mutation accumulation for grapevine and other clonally propagated plants.

## Conclusions

This study described the nature of the mutations causing the diversification of 15 clonally propagated grapevines and confirmed their derivation from a single ancestral mother Zinfandel. The findings indicate that repetitive intergenic space, likely because of its higher rates of methylation in plants, is a significant contributor to the pool of mutations differentially observed among the clones. In addition, the analyses revealed that though comparatively infrequent versus intergenic mutations, relatively large fractions of somatic mutations in exons were deleterious when they were present in individual or a few clones. This result is consistent with the expectation that clones accrue mutations and adds that somatic mutations do not occur uniformly in the genome. These findings add novel insight and nuance to our understanding of the nature and fates of mutations in a clonally propagated organism.

## Methods

### Zinfandel plant material

Fifteen Zinfandel clones were used for this study. Plants were confirmed to be clones of Zinfandel using the following microsatellite markers: VVMD5, VVMD7, VVMD27, VVMD31, VVMD32, VVMS2, VRZAG62, and VRZAG79 [44, 71, 72]. Fourteen of these clones are available through Foundation Plant Services (FPS) at the University of California Davis. Nine of the fifteen clones belong to the Zinfandel Heritage Vineyard Project, a collection of Zinfandel vine cuttings grown in the same vineyard. The identification numbers, common names, and source of the clones used in this study are listed in Table 1. An FPS identification number suffix of “.1” indicates that the clone underwent microshoot tip tissue culture therapy. Pribidrag 13 and Pribidrag 15 are direct cuttings of the mother plants of Pribidrag 4 and Pribidrag 5, respectively, but did not undergo microshoot tip tissue culture therapy. Crljenak kaštelanski 14 did not experience tissue culture; it was also propagated directly from the mother of an FPS accession. Pribidrag 13, Pribidrag 15, and Crljenak kaštelanski 14 are not part of the FPS collection and were retrieved for this study directly from the University of Zagreb. In this manuscript, Zinfandel clones will be referred to by the clone numbers and common names listed in Table 1.

### DNA extraction, library preparation, and sequencing

High quality genomic DNA was isolated from grape leaves using the method described in Chin et al. (2016) [53]. DNA purity was evaluated with a Nanodrop 2000 spectrophotometer (Thermo Scientific, Hanover Park, IL), quantity with a Qubit 2.0 Fluorometer (Life Technologies, Carlsbad, CA) and integrity by electrophoresis. For SMRT sequencing, SMRTbell libraries for the Zinfandel reference FPS clone 03 (Zin03) were prepared as described by Chin et al. (2016). For Illumina sequencing, DNA sequencing libraries for each of the fifteen Zinfandel clones were prepared using the Kapa LTP library prep kit (Kapa Biosystems) as described by Jones et al., (2014) [73]. Final libraries were evaluated for quantity and quality using a Bioanalyzer 2100 (Agilent Technologies, CA). Zin03 SMRTbell libraries were sequenced on a PacBio RS II and Illumina libraries were sequenced in 100 and 150 base-pair paired-end reads on an Illumina HiSeq3000 sequencer (DNA Technology Core Facility, University of California, Davis). Genome sequences of additional *V. vinifera* were used in this study, including long reads from Cabernet Sauvignon (NCBI BioProject PRJNA316730) and short reads from Cabernet Franc, Chardonnay, Merlot, Pinot Noir, and Sauvignon blanc (NCBI BioProject PRJNA527006).

### Zinfandel genome assembly and annotation

De novo assembly of Zinfandel (Zin03) was performed at DNAnexus (Mountain View, CA, USA) using PacBio RS II data and the FALCON-unzip (v. 1.7.7) pipeline [53]. FALCON-unzip was used for its ability to assemble a contiguous, partially phased diploid genome [53, 74]. Repetitive sequences were masked prior to error correction using TANmask and REPmask modules in Damasker [75]. After error-correction (13,073 bp length cut-off), a total of 1.68 million error-corrected reads (N50 15Kbp, 98-fold coverage of expected genome size) were obtained and repeats were masked before overlap detection in the FALCON pipeline (v. 1.7.7). PacBio reads were assembled after testing multiple parameters to produce the least fragmented assembly. These conditions are listed in Additional file 5. Haplotype reconstruction was performed with default parameters. Finally, contigs were polished with Quiver (Pacific Biosciences, bundled with FALCON-unzip v. 1.7.7). Repeats were annotated on the Zin03 assembly using RepeatMasker (v. open-4.0.6) [76] and a *V. vinifera* repeat library [77]. We estimated accuracy by counting the number of non-reference calls for Zin03 Illumina reads mapped to the primary assembly; by this measure, the assembly was 99.92% accurate.

Publicly available datasets were used as evidence for gene prediction (Additional file 5). Transcriptional evidence included *Vitis* ESTs, Cabernet Sauvignon corrected Iso-Seq reads, Tannat, Corvina, and Cabernet Sauvignon transcriptomes, and previously published Zin03 RNA-Seq data. The Swissprot viridiplantae data, TAIR10 *Arabidopsis* data, and *Vitis* data were used as experimental evidence. Each RNAseq sample was trimmed with Trimmomatic (v. 0.36; Additional file 5) and assembled with Stringtie (v. 1.3.3) [78]. A detailed list of all experimental data used for the annotation procedure is in Additional file 5. This data was then mapped on the genome using Exonerate (v. 2.2.0, transcripts and proteins) [79] and PASA (v. 2.1.0, transcripts) [80]. Alignments and *ab initio* predictions generated with SNAP (v. 2006-07-28) [81], Augustus [82], and GeneMark-ES [83] were used as input for EVidenceModeler (v. 1.1.1) [84]. EVidenceModeler was used to identify consensus gene structures using the weight reported in Additional file 5. Functional annotation was performed using the RefSeq plant protein database (ftp://ftp.ncbi.nlm.nih.gov/refseq, retrieved January 17th, 2017) and InteProScan (v. 5) as previously described [77]. Gene space completeness (96.7%) of the final assembly was assessed with BUSCO (v.3) [85].

### Genetic variant calling

Comparisons between Zinfandel clones and between Zin03 and other cultivars were made using the Zin03 genome as reference. This pipeline is described in Additional file 6. Small insertions and deletions (INDELs), single nucleotide variations (SNVs), and structural variations (SVs) were analyzed. The short Illumina reads belonging to the fifteen Zinfandel clones and additional cultivars were trimmed using Trimmomatic (v. 0.36; Additional file 5). Quality filtered and trimmed paired-end reads were then randomly down-sampled to 84 million (29X theoretical mean coverage) in each library to mitigate the possibility of sequencing depth-dependent outcomes. All libraries were aligned to Zin03 using bwa (v. 0.7.10) and the -M parameter [86]. For all genotypes, the median number of reads mapping to the Zinfandel reference genome was 97%. All but one of the Zinfandel clones aligned at greater than 94%; Pribidrag 15 aligned at 80% (Additional file 4: Table S1). The GATK Depth of Coverage tool and read alignments were used to assess coverage on the primary assembly. Taking the average over the Zinfandel clones, sequencing covered 99% of the primary assembly. Next, Picard Tools (v. 2.12.1) were used to mark and filter optical duplicates, build BAM indices, and validate SAM files (http://broadinstitute.github.io/picard). Variants were called using GATK’s HaplotypeCaller (v. 3.5) [87]. Then, called variants were filtered and annotated using GATK’s VariantFiltration tool (DP > 20, DP < 5, QUAL <20, QD < 2.0, FS > 60.0, MQ < 40.0, MQRankSum < − 12.5, ReadPosRankSum < − 8.0). Variant call files were combined using GATK’s GenotypeGVCFs. Having mapped Illumina reads corresponding to the Zinfandel reference onto itself, erroneous non-reference Zin03 calls were removed. This corresponded to 9.9% of the variant calls made among Zinfandel clones. The variants called included SNVs and INDELs. Loci at which all 15 Zinfandel were identically heterozygous (ex. all 0/1) are not considered “variant sites” in this study. Only variant sites at which all samples were called by GATK were included for the construction of figures and tables.

Next, large structural variations between Zin03’s haplotypes, between Zinfandel clones, and between Zin03 and Cabernet Sauvignon (CS08) were studied. Genes annotated on Zin03’s haplotig assembly were mapped to Zin03’s primary assembly to assess differences in gene content between Zin03’s haplotypes using Gmap (v. 2015-09-29) and the following parameters: -K 20,000 -B 4 -f 2. Hits with at least 80% identity and reciprocal coverage were considered matches. Then, SMRT reads from Zin03 were mapped to the Zin03 genome using NGMLR (v. 0.2.7) and structural differences were called with Sniffles (v.1.0.8) [55]. Reads were mapped to the Zin03 primary assembly to examine genome-wide structural variations that may occur between haplotypes. Reads were mapped to the haplotigs specifically to see whether structural variations could account for the Zin03 genes uniquely present in the Zin03 haplotigs. Likewise, Zin03 genes were compared to CS08 by mapping Zin03 coding sequences with Gmap and structural variations were identified in CS08 relative to the Zin03 primary and haplotig assemblies with NGMLR and Sniffles.

Zinfandel clones were compared to one another using Illumina short reads and Delly (v. 0.7.8) with default parameters [88]. The structural variations identified by Sniffles and Delly in Zin03 were intersected. Several filters were applied to the results of SV analyses. Translocations, non-reference Zin03 genotype calls, and SVs annotated at the ends of contigs were filtered from Sniffles and Delly results. In addition, SVs that intersected the repeat annotation were filtered from the Delly results.

### Variant validation

We tested the validity of heterozygous SNVs called by GATK relative to Zin03. A direct comparison between assembled haplotig sequences and the primary assembly was made by mapping with MUMMER4 (ver. 4.0.0, nucmer --mum) [89]. Alignments were filtered with “delta-filter” tool of the same suite (default parameters), followed by SVs calling with “show-snps” (−Clr -× 1) and “show-diff” (default parameters). Variant calls were also made with an additional tool and using long PacBio reads generated with a different sequencing technology. These were mapped with Minimap2 (ver. 2.16, −ax map-pb --MD --cs -L) [90] and variants called with Clairvoyante (downloaded March 26, 2018; −-threshold 0.2 --minCoverage 4 --threads 8 --chkpnt_fn Clairvoyante/trainedModels/fullv3-pacbio-ngmlr-hg001 + hg002 + hg003 + hg004-hg19/learningRate1e-3.epoch100.learningRate1e-4.epoch200) [91]. The variant sites considered for cross-validation between methods were those (i) in non-repetitive regions (ii) that were covered by haplotig mapping with MUMMER4. Nearly half of the variant sites (717,647 out of 1,446,289 positions) were called by all three methods (Additional file 3: Table S1). Only 8.9% of the variant sites called by GATK were not called by either of the other methods and 6.9% of all possible variant sites were uniquely called by GATK (Additional file 3: Table S1). In addition to this validation of the variant calling pipeline, ten selected variants called in five genes were also validated with ~ 80% success by Sanger sequencing. The genes within which these variants occurred, the locus of the variant, and the primer sequences used are listed in (Additional file 3: Table S2).

### Transposon insertion analysis

PoPoolationTE2 (v. 1.10.04) [92] was used to identify transposon insertions in the Zinfandel clones; it was used following the workflow outlined in its software manual (https://sourceforge.net/p/popoolation-te2/wiki/Manual/). Insertions were called relative to PN20024 [54]. As described in Kofler *et al.* (2016), PoPoolationTE2 analyses transposable element insertions and can identify novel and annotated TE insertions provided insertions fall within predefined families of TEs. In this manuscript, the TE insertions among the clones are reported using the classification system and nomenclature described by Wicker *et al.* (2007) [93]. In instances where the TE order and/or superfamily was not annotated, only the TE class and order, when available, are named in the associated figures and text.

### Relationships between zinfandel clones

The relationships between Zinfandel clones were visualized by Principal Component Analysis and their relatedness was quantified (VCFtools v. 0.1.15) based on the method described by Manichaikul et al. (2010) [56]. This approach gives information about the relationship of any pair of individuals (unrelated, 3rd degree relative, 2nd degree relative, full siblings, and self) by estimating their kinship coefficient, which ranges from zero (no relationship) to 0.50 (self).

## Supplementary information

**Additional file 1:** Method to extraction phenolic metabolites from Heritage Vineyard Zinfandel clones and discriminant analysis of Zinfandel clones based on their phenolic profiles.

**Additional file 2:** Zinfandel genes intersecting structural variations identified between haplotypes (4459) with associated Gene Ontology categories.

**Additional file 3:** Results of technical (Table S1, Figure S1) and Sanger sequencing (Table S2) validation of variant calls by GATK.

**Additional file 4: Table S1.** a summary of the alignment of Illumina libraries to Zin03, **Table S2.** a summary of variants relative to the Zinfandel primary assembly, and **Table S3.** a summary of the SnpEff analysis of variants, with mean values ± SEM shown.

**Additional file 5:** Settings and data used for Zin03 genome assembly, annotation, variant calling, and validation procedure.

**Additional file 6:** Bioinformatic pipeline for SNV, INDEL, and SV calling.

## Data Availability

The datasets supporting the conclusions of this article are available in two locations. Raw sequences are available at NCBI (Bioproject PRJNA527006). Other relevant data, such as genome sequence, gene and protein sequences, gene and repeat coordinates and annotation, along with a genome browser and a blast tool, are available at http://cantulab.github.io/data.html.

## Abbreviations

CS08: Cabernet Sauvignon 08
FPS: Foundation Plant Services
INDEL: Insertion/Deletion
PCA: Principal Component Analysis
SMRT: Single Molecule Real-Time
SNV: Single Nucleotide Variant
SV: Structural variant
TEI: Transposable element insertions
UC: University of California
ZAP: Zinfandel Advocates and Producers
Zin03: Zinfandel 03
