# Supplementary material for: The genomic diversification of grapevine clones
Source: BMC Genomics. 2019 Dec 12;20:972. doi: 10.1186/s12864-019-6211-2 (PMC6907202; doi:10.1186/s12864-019-6211-2)
Supplement: Supplementary file 1 — Additional file 1: Method to extraction phenolic metabolites from Heritage Vineyard Zinfandel clones and discriminant analysis of Zinfandel clones based on their phenolic profiles. [file 12864_2019_6211_MOESM1_ESM.pdf]

Additional file 1

Extraction of Phenolic Compounds and HPLC-DAD

Phenolic acids, cinnamic acid, flavanones, and anthocyanins in seven clones from the Heritage Zinfandel Collection (Clones 1 and 6-11) were measured by HPLC-DAD. Four biological replicates were sampled at commercial harvest (~23-26 ºBrix) and technically duplicated for all but one of the clones. Four biological replicates were taken of Clone 6, but one of them was not technically duplicated. Berry skins were blended for three minutes, mixed with 1L of solvent (1M HCl in 95% ethanol), placed in a boiling water bath for 20 minutes, and allowed to cool for one hour at room temperature. The solution was filtered through a 0.45-µm PTFE membrane prior to HPLC-DAD.

An Agilent 1100 HPLC system (Agilent Technologies, Santa Clara, CA) with a diode array UV-visible detector coupled to an Agilent ChemStation (Rev. A.10.02) and 5 µm ChromoSpher RP-18 column (Agilent Technologies) were used for solvent delivery and detection. The flow rate was 0.5 mL/min, Solvent A was 50 mM dihydrogen ammonium phosphate adjusted to pH 2.6 with orthophosphoric acid, Solvent B was 20% A in 80 % acetonitrile, and Solvent C was 0.2 M orthophosphoric acid adjusted with ammonia to pH 1.5. Separations were performed at 40°C and signals were recorded at 280 (Phenolic acids), 316 (Cinnamic acid), 365 (Flavanones), and 520 (Anthocyanins) nm. ChemStation was used to identify and quantify metabolite classes.

Additional file 1

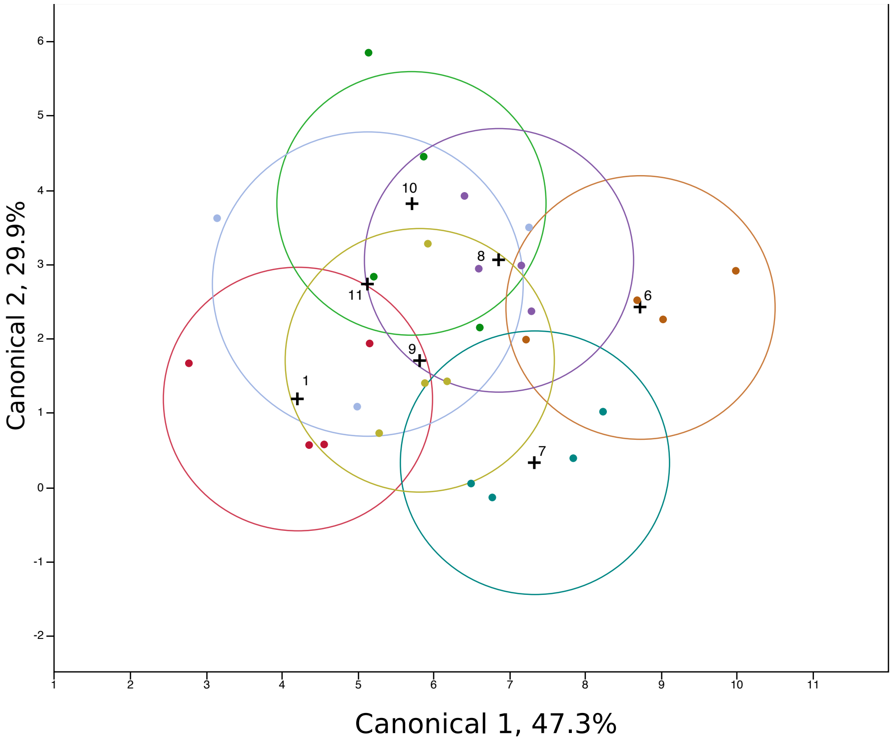

Linear discriminant analysis of seven Zinfandel selections using their phenolic content (Phenolic acids, cinnamic acid, flavanones, and anthocyanins) measured by HPLC. 95% Confidence intervals for each clone are shown. These subtle differences could reasonably be attributed to biological differences because these fruits were sampled from a common, uniformly managed vineyard. However, additional studies are necessary to determine the genetic basis of these differences, whether they lead to detectable sensory differences, whether they are reproducible across vintages, and comprehensively profile the chemical and morphological differences among clones.
